# 

**Published:** 1876-04

**Authors:** 


APRIL, 1876.

### THE AMERICAN JOURNAL OF DENTAL SCIENCE,

EDITED BY

F. J. S. GORGAS, M. D., D. D. S.

VOL. 9.—THIRD SERIES.—No. 12.

PUBLISHED MONTHLY.

$2.50 PER ANNUM.

BALTIMORE:

SNOWDEN & COWMAN.

LONDON:

TRUBNER & CO., 60 PATERNOSTER ROW.

1876.

PAYABLE IN ADVANCE.

